# Determination of Cleaning Limits Considering Toxicological Risk Evaluation to Minimize the Risk of Cross Contamination

**DOI:** 10.22037/ijpr.2020.112734.13922

**Published:** 2021

**Authors:** Somayeh Lamei Ramandi, Ramin Asgharian

**Affiliations:** a *Department of Analytical Chemistry, Faculty of Chemistry, Islamic Azad University, North Tehran Branch, Iran. *; b *Department of Pharmaceutics, Faculty of Pharmacy, Islamic Azad University of Pharmaceutical Sciences Branch, Tehran, Iran.*

**Keywords:** Cleaning validation, Contamination, Exposure, Swab, Risk

## Abstract

The historical approaches that have been used to establish cleaning validation acceptance limits should be updated to recent approaches to prevent cross contamination. In the present investigation, a cleaning method was validated using high performance liquid chromatography. Method modification critical parameters including spiking, swab sampling from PVC, Stainless Steel, and Polyethylene, extraction technique from swab, solubility, potency, toxicity (LD50), and improvement of limit of detection (LOD) of the method through analytical method validation were studied. In addition, roughness, mechanical and electro-polishing, consideration of dosage form as a quantitative factor, acceptable daily exposure (ADE), and permitted daily exposure (PDE) in the worst-case determination were considered in the study. The method was validated based on USP and ICH guidelines for specificity, limit of detection, limit of quantitation, precision, accuracy, linearity, and range. Linear regression analysis of data for the calibration plot in the range of 7.43, 10.89, 21.78, 43.56, 87.12 µg/mL, and relative standard deviation (R.S.D.) found to be 0.5, 0.4, 0.2, and 0.2, respectively with correlation coefficient of R^2^ = 0.999997. The LOD and the limit of quantitation (LOQ) were 2.23 and 7.43 µg/mL, respectively. Good recoveries in the range of 73.65-81.20%, and precision with relative standard deviation values lower than 15% have been obtained. The proposed method developed for cleaning validation is specific, precise, and useful for determination of cleaning acceptance limits using health-based limit and Quality Risk Management to develop an appropriate cleaning program for engineering design, safety of patients, and worker protection.

## Introduction

Old and new approach for determination of cleaning validation acceptance limits:

Cross contamination is one of the highest risks for patients using pharmaceutical products (1-4). Due to the perceived risk, certain classes of medicinal products have been previously required to be manufactured in dedicated or segregated self-contained facilities including, *“*certain antibiotics, certain hormones, certain cytotoxics ,and certain highly active drugs” (1).

There have been no standardized definitions in terms of what “certain” means and which subset of compounds in each class requires dedicated facilities. Since the new pharmacological and toxicological mechanisms challenge these categories, it is required to modify the historical use of these terms to identify and prioritize compounds requiring special attention (1, 4 and 5).

The technical and organisational deficiencies including insufficient cleaning of equipment, poor facility design, or inappropriate design of the HVAC system are the main reasons for cross contamination (4). 

The formerly accepted methods for establishment and calculation of cleaning validation acceptance limits including visually clean, 0.1% dose, and 10-ppm criteria have become obsolete (6). Recent update to the European Union regulation on GMP parts 3 premises and equipment (7) and chapters 5 production (2) show that assessing risk to manage cross contamination and cleaning should be dependent on a toxicological assessment to set threshold, or acceptable daily exposure (ADE) values with the focus on minimizing the risk of cross contamination (1). 

According to Paracelsus (1493–1541), the father of toxicology, “all substances are poisons; there is none which is not a poison”. The right dose differentiates poison from a remedy” (8). The acceptable daily exposure approach is inherent in fundamental principles of toxicology. According to the International Society for Pharmaceutical Engineering (ISPE), ADE is “an unlikely dose bringing about a negative effect in an individual upon exposure to, by any route, this or less than this dose daily for a lifespan” (4). According to the European Medicines Agency (EMA), the permitted daily exposure (PDE) is “an unlikely substance-speciﬁc dose bringing about ‎a negative effect in an individual upon exposure to this or less than this dose daily for a lifespan” (1). Adverse effect is undesirable and unintended, although not necessarily unexpected result of therapy or other intervention (9, 10).

European Medicines Agency (EMA) guideline and ISPE Risk-MaPP Baseline Guide focus on the use of quality risk management (QRM) on compound-specific Acceptable Daily Exposure to calculate the limits for the maximum carryover by taking into account the toxicological and pharmacological properties of each single product, Occupational Exposure Limit (OEL), and movement away from putting compounds into categories (1, 4, 11 and 12). 

Although criteria for Industrial Hygiene (IH) and current Good Manufacturing Practices (cGMP) originate from the same toxicological or clinical data, differences are applied to use these data to set acceptable limits and adequate control strategies.

Differences between IH and cGMP considerations perspectives include worker and product exposure, route of entry, exposure mechanism, and basics of standards for risk assessment (4, 11 and 12). Table 1 summarizes IH and cGMP considerations for risk assessment. 

In order to prevent cross contamination, it is necessary to have an overview on the process map and identify IH and cGMP requirements in each process and relevant sub processes. Table 2 shows exposure potential risk assessment based on dustiness, quantity, task duration. Table 3 describes cross contamination risk reduction, considering occupational exposure band (OEB), occupational exposure level (OEL), and containment level (4, 11 and 12).

In order to obtain the appropriate criteria to support cleaning, it is necessary to determine the acceptable level of retention or carryover from one product on a case-by-case basis. The hazard(s) created by the product, the nature and route of administration of the product should be processed in the equipment, unit, or facility. A suitable safety margin, alert and action levels should be incorporated when establishing acceptable levels. Factors for evaluating are feasibility, practicability, cost, and patient, if the cleaning criteria can be met or detected. By dealing with these factors satisfactorily, a multi-product facility could be a viable option to process or make the target material or product (4). 

Based on the results of a risk assessment, segregation and dedication may also be required for some compounds to prevent cross contamination during pharmaceutical products manufacturing (4, 13 and 14). 

## Experimental

*Materials and Methods*

HPLC grade of Acetonitrile (ACN) was purchased from Romil (UK). Triethylamine, and Trifluoroacetic acid were obtained from Merck (Germany). HPLC grade water was obtained in-house from Puris Expe-UP water purification system (Korea). Gliclazide BPCRS was used as the reference standard. 

The Alpha swabs (Texwipe® 761) used for the cleaning assessment were from Texwipe (Philippines). The coupons with surfaces similar to the manufacturing equipment were constructed in house from Stainless Steel, Polyvinyl Chloride (PVC), and Polyethylene (PE) with dimensions of 5 cm × 5 cm. The internal material finish should have an arithmetical average surface roughness of no longer than 0.8 micrometer (15). The Stainless Steel coupons were electro-polished and the average roughness was 0.074 µm. In order to increase reproducibility in the recoveries, to increase the recovery results and to reduce the variability, it is important to regenerate the test surfaces back to the original state. Appropriate cleaning the surface of the coupons is of tremendous importance to perform the quantitative swabbing process successfully. The coupons were ultrasonicated in water, rinsed with purified water, and dried at ambient temperature.

*Instrument*

Throughout the measurements, a Waters Alliance series HPLC system involving Waters e2695 Separations Module, and UV-VIS detector model 2489 was employed (USA). The system was fitted with a pump, auto-sampler, and Thermostatic Column Compartment (TCC). Empower 3 software was applied for gathering HPLC data and processing. Operating conditions of HPLC in this study are summarized in Table 4.

*Standard preparation*

Stock standard solution of Gliclazide (0.2 mg/mL) was prepared by weighing approximately 40 mg of Gliclazide BPCRS into a 200 mL volumetric flask, dissolving it in 10 mL of Acetonitrile, and diluting to 200 mL using a mixture of 2 volumes of Acetonitrile and 3 volumes of Water.

*Method validation*

Method validation requirements depend on the intended use of the method, the status of the method, and the development stage of the product (16-19). 

*System suitability*

Verification of the appropriate performance of the overall system composing of instrument and method at the time of use is regarded as an integral part of the analytical procedure. System suitability test (SST) verifies the actual suitability each time the analytical procedure is applied and can be regarded as a part of continued method performance verification. SST should be designed to detect variation in the performance of a procedure in routine use and it should be based on an understanding of the risk and impact of variation with the acceptance criteria chosen to ensure that the measurement uncertainty remains acceptable (17). SST solution was prepared by dissolving 5 mg of Gliclazide BPCRS and Gliclazide impurity F BPCRS in 25 mL of Acetonitrile, diluting to 50 mL with water and subsequent dilution of 1 volume of the resultant solution to 20 volumes with a mixture of 45 volumes of Acetonitrile and 55 volumes of water (20).

*Linearity and Range*

Linearity of an analytical procedure is its capability within the target range to yield test results that are directly proportionate to the concentration of analyte in the sample. Range is the span between the higher and lower concentration of analyte in the sample according to which the analytical procedure has an appropriate level of precision, accuracy, and linearity (17-19). Linearity of the method was studied by determining standard solutions at five different concentration levels. 

*Accuracy*

Accuracy of an analytical procedure expresses the closeness of agreement between the value, which is accepted as either a conventional true value or an accepted reference value and the value found. This is sometimes termed “trueness” (18, 19). The uncorrected bias or systematic error in an analysis is measured by accuracy (17). It is necessary to report accuracy as percent recovery by the assay of known added amount of analyte in the sample or as the discrepancy between the mean and the established true value together with the confidence intervals. It is also necessary to assess accuracy using a minimum of 9 determinations over a minimum of 3 concentration levels covering the specified range and 3 replicates each (18). There should be evidence that the samples are accurately recovered. A recovery of >80% is considered good, >50% reasonable, and <50% questionable (21). 

*Precision*

Precision of an analytical procedure is the degree of agreement among individual test results when the procedure is applied repeatedly to multiple samplings of a homogeneous sample. The precision of an analytical procedure is usually expressed as the variance, standard deviation, or coefficient of variation of a series of measurements (18, 19). 

*Connection between Accuracy and Precision*

Precision and accuracy, as the two key components, can be considered together to show if the total error is within the boundary conditions of the material acceptance criteria. Increased precision of a procedure makes any bias inherent in the system more obvious, sheds light on the linearity and range, makes the capability of determining an error brought about by a lack of specificity more obvious. It also enhances the probability of a material meeting the assay acceptance criteria receiving a passing result. Precision and accuracy are the most meticulously connected of these relationships (17).

*Specificity*

Specificity is the ability to assess the analyte in the presence of components, which may be expected to be present. Typically, these might include impurities, degradation products, and matrix components (18, 19). 

*LOD and LOQ*

The minimum detectable amount of analyte in a sample that cannot be necessarily quantitated under the stated experimental conditions is called Limit of detection (LOD). ‎

Limit of quantitation (LOQ) is the minimum‎ amount of analyte in a sample that can be determined with acceptable precision and accuracy under the stated experimental conditions. 

*Sample preparation*

Defined volume of sample solution at a specific concentration was loaded on 5cm×5cm pre-defined surfaces. The coupons were left to dry at room temperature. A dry swab was pre-wetted with water for wiping the surface of the coupon up to the total 25-cm^2^ test area.

Four edges of coupon were also wiped. The swab head was cut and put into the premeasured solvent volume in the vial. The process was performed over again by a second swab. Two swabs were put in each vial. The vial was sonicated for five minutes; then, the solution was transferred to an HPLC vial and labeled as sample solution.

*Adjusting Health-Based Safety Thresholds/Acceptance Limits*

The establishment of acceptance limits is necessary for cleaning the validation programs. Setting the limits requires the right margin of safety. The “Margin of Safety” by definition is the distance of the cleaning data from the acceptance limit. Adjusting the acceptance criteria to a health-based limit such as the ADE leads to several enhancements. 

The ADE is derived based on toxicology and not just dosage. The ADE is a properly adjusted “safe level” to clean residues. Operationally, the ADE makes it possible to estimate the true “Margin of Safety” upon the assessment of cleaning residue data. It is made sure that any residuals following cleaning are as low as possible below the health-based criteria, and reducing the risk of cross contamination is possible by assessing the cleaning validation data. It is noted that ‎the ADE is not a “limit” in the true sense. However, it is a reference point to determine the level of “risk” made by the residue data. It is believed that acceptable daily exposure and permitted daily exposure are synonyms, but with the emphasis that specific terms are selected by various regulatory bodies. 

It is possible to derive ADE from the following Formula (4):

$(\mathrm{ADE})=\frac{\mathrm{NOAEL}(\mathrm{mg}/\mathrm{kg}/\mathrm{day})\times \mathrm{BW}(\mathrm{kg})}{\mathrm{UFc}\times \mathrm{MF}\times \mathrm{PK}}$

Where: NOAEL shows the no observed adverse effect level, BW is body weight, UFc shows the composite uncertainty factor, MF shows the modifying factor, PK is the Pharmacokinetic Adjustment(s). 

PDE can be derived from the following Equation (1):

$(\mathrm{PDE})=\frac{\left(\mathrm{NOAEL}\times \mathrm{Weight Adjustment}\right)}{\mathrm{F1}\times \mathrm{F2}\times \mathrm{F3}\times \mathrm{F4}\times \mathrm{F5}}$

Where: NOAEL shows the no observed adverse effect level and F1 to F5 deal with different forms of uncertainty.

## Results and Discussion

*Method validation*

The method was validated for the critical validation parameters (CVPs).

*System suitability*

The system suitability test is not valid, unless, in the chromatogram obtained, the resolution factor between the peaks due to Gliclazide and Gliclazide impurity F is at least 1.8. The resolution found to be 2.03

*Linearity and Range*

**Figure 1:**
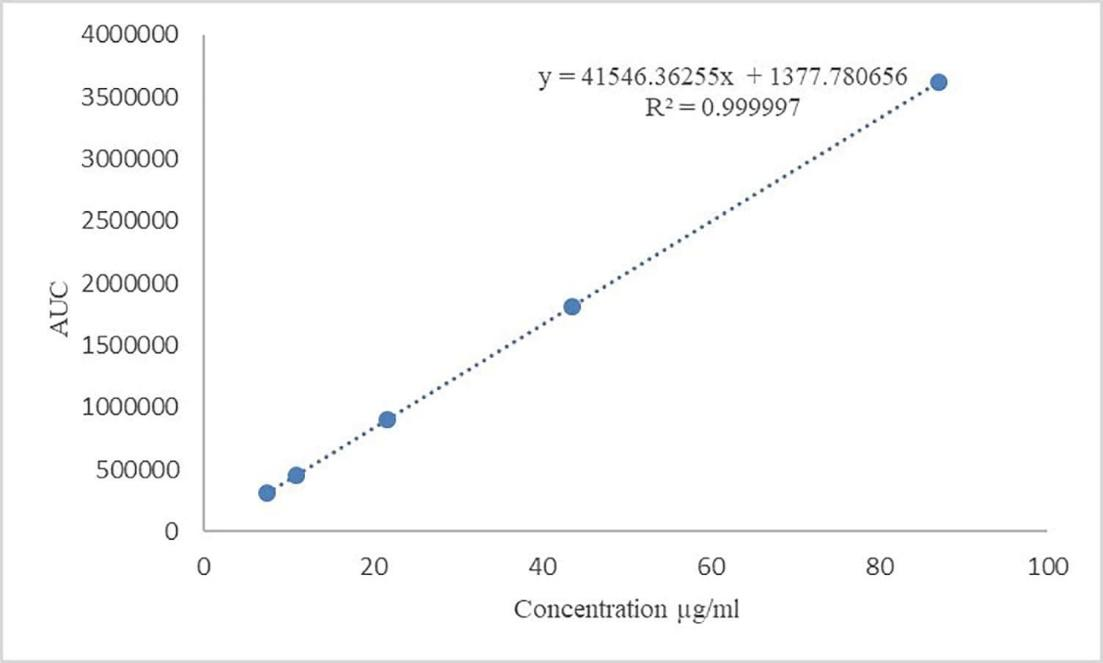
Calibration curve of Gliclazide with different concentration levels

The result of the standard curve, which was constructed by the least square regression method, is presented in Table 5. Linearity calibration curve is shown in Figure 1, which indicates that the procedure is capable of yielding accurate and precise results at any concentration to measure the target material. The standard stock solution of Gliclazide was diluted to concentration containing 7.43, 10.89, 21.78, 43.56, and 87.12 µg/mL Gliclazide. All solutions were injected in triplicate. Mean found to be 310142, 455426, 906887, 1807240, 3622481 and standard deviations were 2531, 2451, 3452, 3163, and 7330, respectively.

*Accuracy*

**Figure 2:**
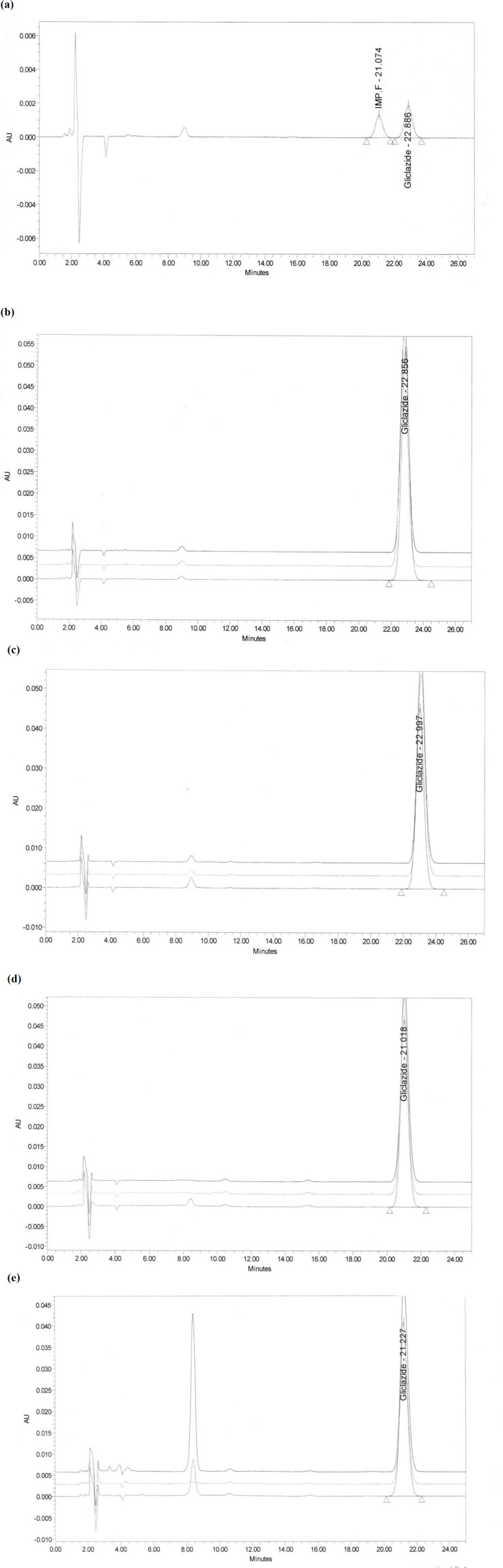
Chromatograms of recovery studies at 100% concentration level of Gliclazide swab sampling: (a) System suitability; (b) Standard solution; (c) PVC; (d) Stainless Steel; (e) Polyethylene

Accuracy test results revealed that the recovery of reference material covering the specified range including 50, 100, and 200% concentration levels, which were spiked to 5 cm × 5 cm coupons, were in accordance to the acceptable criteria. As shown in Table 6, the proposed method was highly accurate for the quantitative analysis. Figure 2 illustrates chromatograms of system suitability, standard, and recovery studies at 100% concentration level of Gliclazide swab sampling from PVC, Stainless Steel, and Polyethylene.

*Precision*

Precision was determined by assaying a homogeneous sample using nine determinations covering the specified range of the procedure for each pre-defined surface. Table 7 indicates acceptable precision, R.S.D. lower than 15%, across the entire dataset.

*Specificity*

Specificity was performed by spiking product with appropriate level of excipients. Specificity test results proved that all excipients could be separated completely.

*LOD and LOQ*

The LODs (S/N = 3) and LOQs (S/N = 10) were found to be 2.23 µg/mL and 7.43 µg/mL, respectively, based on the analytical method validation of assay.

For mitigating the risk of cross contamination based on the failure mode and effect analysis (FMEA), more restrictive acceptance limit through adding safety factors to cleaning validation acceptance criteria was considered as preventive control. Obtained results showed that this approach only leads to failed results and costly measures in production operations. The better developed cleaning process, focusing on decreasing the remained residue in production instead of adding safety factors to acceptance limits leads to the main goal, which is patient safety.

The initial attempts for recovery studies revealed low and changeable recoveries from coupon surfaces. Ishikawa diagram was used to break down root causes that potentially contribute to failure in recovery test results as a defect. Method modification was studied for obtaining consistent and high recovery. Critical parameters were swabbing, spiking, and extraction technique of the API from swab. None of the parameters eliminated the previously observed variability. The major contributor to low and variable recoveries from coupons was traced to the lack of standard operating procedure for cleaning the coupon surfaces. 

Material investigation was done to study if the coupon was a source of inconsistency. Roughness test was performed on both the coupons and drums. In order to improve the resistance of the stainless-steel material and prevent corrosion and improper cleaning of the surface of the coupons in recovery studies and production equipment, mechanical and electro-polishing were employed on the surfaces which were not meeting roughness acceptance criteria. 

Variable recoveries from stainless steel coupons in different spiking levels including 50, 100, and 200% were studied. It was observed that spiking at the lower concentration levels displayed not acceptable recoveries due to experimental errors on recovering lower mass in comparison to the larger mass of the drug. The precision results in Table 7, with R.S.D. lower than 15% indicates that preventive and detection controls, based on the FMEA, including analyst training, following standard operating procedure, and monitoring the process were effective. 

Sampling and dispensing tools with very low risk of cross-contamination were considered disposable and dedicated for the worst cases based on the result of the risk assessment. Although the cleaning validation results were within the limit leading to extra costs to have added safety to the patient, they were unjustifiable by measurable means. 

Assessment of the worst case of the cleaning validation was done based on solubility, potency, toxicity (LD50), ADE, PDE, improvement of LOD of the method through analytical method validation, and consideration of the dosage form. The higher risk of cross-contamination of uncoated tablets compared to coated tablets is a quantitative factor to increase the margin of safety. 

Cleaning acceptance criteria based on the MAC approach and 10 ppm were 16390 and 672 mg, respectively. Ten ppm was utilized as the worst case to establish the limit. The highest dosage of Gliclazide was defined as the worst case. Gliclazide 80 mg was selected as the worst case among Gliclazide 80 mg, 60 mg, and 30 mg. 

**Table 1:** Standards for risk assessment, Industrial Hygiene and cGMP considerations (8).

**Industrial Hygiene (IH)**	**Quality (cGMP)**
Occupational Exposure Limit (OEL) expressed by an airborne concentration (mass per cubic meter of air) to address primary route of entry for exposure: Inhalation	Acceptable Daily Exposure (ADE) expressed as mg/day
	Cleaning Limit expressed as mg/swab or mg/L to address primary route of exposure: Ingestion, 4

**Table 2:** Defining factor of the quantity of loose powder transfer considering dustiness potential, quantity handled, and task duration in exposure potential risk assessment (4, 11 and 12)

**Factor**	**Level**		
Dustiness potential	Low	Medium	High
Quantity handled	Small	Medium	High
Task duration	Short	Medium	Long

**Table 3:** Reducing the risk of cross-contamination by airborne or mechanical transfer routes considering occupational exposure band (OEB), occupational exposure level (OEL), and containment level (4, 11 and 12).

**Occupational exposure band (OEB)**	**Occupational exposure level (OEL)**	**Containment level**
5	< 1 µg/m^3^	High
4	1 to 10 µg/m^3^	
3	10 to 100 µg/m^3^	
2	100 to 1000 µg/m^3^	
1	1000-5000 µg/m^3^	Low

**Table 4:** Chromatographic conditions

Column	Zorbax Eclipse
	C8, Octylsilyl silica gel
	250 mm × 4.6 mm, 5 µm
Mobile phase	0.1 volume of Triethylamine
	0.1 volume of Trifluroacetic acid
	45 volumes of Acetonitrile
	55 volumes of Water
Flow rate	0.9 mL/min
Injection volume	20 µL
Detector UV-VIS	235 nm
Column temperature	Ambient

**Table 5:** Statistical data of calibration curve of Assay

Analyte (Gliclazide)	
**Regression equation**	y = 41546.36255x + 1377.780656
**Linearity range**	7.43-87.12 (µg/mL)
**y-intercept**	1377.780656
**Slope**	41546.36255
**Correlation coefficient**	0.999997

**Table 6:** Accuracy by recovery of the chromatographic method

**Material substance of product contact surfaces**	**Sampling Method**	Concentration Levels		
		50 (%)	100 (%)	200 (%)
Stainless Steel	Swab	95.30	84.84	81.20
Polyvinyl Chloride (PVC)	Swab	88.21	92.50	73.65
Polyethylene (PE)	Swab	69.56	72.52	74.93

**Table 7:** Precision by recovery of the chromatographic method

**Material substance of product contact surfaces**	**Sampling Method**	**Concentration Levels**		
		**50 (%)**	**100 (%)**	**200 (%)**
Stainless Steel	Swab	3.66	4.02	7.26
Polyvinyl Chloride (PVC)	Swab	1.94	6.69	3.85
Polyethylene (PE)	Swab	5.15	9.94	5.47

## Conclusion

This study demonstrates that the cleaning method is validated for specificity, limit of detection, limit of quantitation, precision, accuracy, linearity, and range. Risk assessment for Industrial Hygiene (IH) and cGMP was done science based criteria were defined for making decision to manage the risk to the patients and worker protection.
